# What is the physiological impact of reducing the 2,000 m Olympic distance in rowing to 1,500 m and 1,000 m for French young competitive rowers? Insights from the energy system contribution

**DOI:** 10.3389/fphys.2022.896975

**Published:** 2022-07-18

**Authors:** Allison Diry, Sébastien Ratel, Alan Nevill, Hugo Maciejewski

**Affiliations:** ^1^ French Rowing Federation, Paris, France; ^2^ AME2P—EA 3533, Clermont-Auvergne University, Clermont-Ferrand, France; ^3^ Faculty of Education, Health and Wellbeing, University of Wolverhampton, Walsall Campus, Walsall, United Kingdom

**Keywords:** adolescent, allometric modeling, body mass, aerobic, anaerobic

## Abstract

French rowing federation reduced the competition distance to 1,500 and 1,000 m in rowers under 16- (U16) and 14-year-old (U14) respectively, to prepare them progressively to the Olympic 2,000 m distance in under 18-year-old (U18). This study aimed to check the hypothesis that relative aerobic (%E_Ae_) and anaerobic (%E_An_) energy contributions would be comparable between the competition distances since the more oxidative profile of younger age categories could offset the greater anaerobic contribution induced by shorter rowing races. Thirty-one 12- to 17-year-old competitive rowers performed a race of 2,000, 1,500, or 1,000 m on a rowing ergometer according to their age category. %E_Ae_ and %E_An_ were estimated from oxygen consumption, changes in blood lactate concentration and their energy equivalents. %E_Ae_ was lower in U16 than U18 (84.7 vs. 87.0%, *p* < 0.01), and in U14 than U16 (80.6 vs. 84.7%, *p* < 0.001). %E_An_ was higher in U16 than U18 (15.3 vs. 13.0%, *p* < 0.01), and in U14 than U16 (19.4 vs. 15.3%, *p* < 0.01). The results did not confirm our initial hypothesis since %E_Ae_ and %E_An_ were significantly different between the race distances, and thus age categories. However, %E_An_ in U18, U16 and U14 were found to be in the range of values previously found in adult rowers over the 2,000 m Olympic distance (12–30%). Therefore, on a practical level, the strategy implemented by the French rowing federation to reduce the competition distance in the younger age categories could be relevant to progressively prepare them to the physiological requirements encountered over the Olympic distance.

## Introduction

Olympic rowing events are conducted over a 2,000 m race and last from about 5 min 20 s to 7 min, depending upon the number of rowers in the boat, competition classification, and environmental conditions. Rowing performance is positively associated with the maximal capacity of aerobic and anaerobic pathways to supply energy in exercising muscles (Hagerman et al., 1978; Hagerman, 1984; Secher, 1993; Steinacker, 1993; Pripstein et al., 1999). While the maximal capacity to supply aerobic energy during exercise can be easily determined from the measurement of maximal oxygen consumption (V̇O_2max_), no “gold standard” method currently exists to assess anaerobic contribution (Zwiren, 1989). However, based on an energy equivalent estimation of lactate accumulation in blood, di Prampero (di Prampero, 1981; di Prampero and Ferretti, 1999) proposed an original method to assess the activity of glycolytic metabolism during exercise. From this approach, de Campos Mello et al., 2009 estimated in well-trained adults that the aerobic contribution was ∼84% of total energy expended during a 2,000-m rowing race; the remaining 16% being supplied by the anaerobic pathways. Other studies also report aerobic and anaerobic relative contributions between 70 and 88% and between 12 and 30% respectively, using the methods of accumulated oxygen deficit (Pripstein et al., 1999; Russell et al., 2000) and excess post-exercise oxygen consumption (Hagerman et al., 1978; Secher, 1983). However, despite the relatively small contribution of anaerobic pathways into total energy supply during a 2,000 m rowing ergometer performance, elite adult rowers exhibited high post-exercise veinous blood lactate concentration (up to 32 mmol L^−1^) associated with low blood pH (as low as 6.74) (Nielsen, 1999).

To progressively prepare young competitive rowers for the physiological requirements encountered over the Olympic 2,000 m distance, the French rowing federation has chosen to limit exercise duration by reducing the competition distance. The boat race distance is reduced to 1,500 m in rowers under 16-year-old (U16) and to 1,000 m in rowers under 14-year-old (U14); rowers under 18-year-old (U18) and beyond competing over the 2,000 m Olympic distance. This approach to reducing competition distance in young athletes has also been utilized in other sporting disciplines such as athletics, cycling, triathlon and cross-country skiing. However, in adults, a reduced exercise duration associated with a higher exercise intensity is well known to increase the relative energy contribution derived from anaerobic metabolism into total energy turnover (Gastin et al., 1995; Duffield et al., 2005; Zouhal et al., 2012), thereby departing from the relative energy ratio between aerobic and anaerobic pathways during the 2,000-m Olympic distance (i.e., ∼85/15%). This response could be different in younger age categories since children rely more on oxidative than anaerobic metabolism during exercise than adults (Ratel and Blazevich, 2017). This greater relative aerobic contribution in peripubertal athletes could then offset the greater relative anaerobic contribution induced by shorter but potentially more intense rowing races, and thereby make the relative energy contributions equivalent between the three race distances (i.e., 2,000, 1,500 and 1,000 m) or age categories (i.e., U18, U16 and U14). However, this age-adjusted competition distance approach and the relative concurrent effects of age and race distances remain to be quantified.

In addition, previous studies that investigated anaerobic metabolism during exercise in children did not factor dimensional changes into their data interpretation (Fellmann et al., 1988; Falgairette et al., 1991; Mero et al., 1991). However, total working muscle mass is associated with lactate production (Jensen-Urstad et al., 1994) and accumulation (Volianitis et al., 2018) and *in fine,* could influence relative aerobic and anaerobic energy contributions, as lactate is a key parameter of this estimation. In addition, using allometric scaling, Diry et al. (2020) showed that body dimensions might have a more powerful influence than maturity status on anaerobic metabolism in young competitive rowers. Thus, the smaller body mass (BM) of young rowers could trigger less anaerobic metabolism during exercise, but this response could be counterbalanced by a greater anaerobic contribution due to shorter and potentially more intense races in the U14 and U16 categories.

Therefore, the purpose of the present study was to compare the relative energy contributions derived from aerobic (%E_Ae_) and anaerobic (%E_An_) pathways into total energy production between the 2,000 m in U18 rowers, 1,500 m in U16 rowers, and 1,000 m in U14 rowers. We hypothesized that %E_Ae_ and %E_An_ would be comparable between the three race distances, and thus age categories. A simple allometric model will be used to consider the concurrent effects of BM on E_Ae_ and E_An_ among the three race distances (or age categories).

## Methods

### Experimental approach of the problem

The experiments were conducted on two sessions carried out in a controlled laboratory setting on 2 days apart at the same time of day. Participants were instructed not to undertake any strenuous activity during the 24 h preceding each session. The first session was dedicated to gathering participants’ physical characteristics (anthropometric measurements) and V̇O_2max_ assessment. Then, during the second session, the rowers covered a race distance of 2,000 m for U18, 1,500 m for U16 and 1,000 m for U14, on a rowing ergometer (Model D, Concept2, Morrisville, VT, United States A) as fast as possible, according to the recommendations of the French rowing federation. All the rowers were already fully familiarized with the equipment. The computer of the ergometer continuously delivered the power output (in W). The investigators set the resistance factor between 100 and 130 according to age and the expertise level of young rowers. The same resistance factor was used for the two exercise sessions. Verbal encouragement was systematically provided by the investigators during each exercise session.

### Participants

Thirty-one male competitive rowers aged from 12 to 17 years volunteered to participate in the present study. They were classified into three age categories 1) under 18 years (U18: 16.0–17.9 years, *n* = 9), 2) under 16 years (U16: 14.0–15.9 years, *n* = 10) and 3) under 14 years (U14: 12.0–13.9 years, *n* = 12). For the whole population, all tests were carried out between October and March, i.e., during the winter training period, before the start of the competitive season in May. All participants trained 3 to 6 times per week (i.e*.*, 2–4 “on-water” training sessions and 1–2 physical training sessions) in the year preceding the experiments and have already participated in regional or national competitions. None of the participants had a family history of cardiovascular disease or was using any medication. The present study was approved by an institutional ethics review board (Comité d'Éthique pour la Recherche en Sciences et Techniques des Activités Physiques et Sportives—CERSTAPS, n° 2017-29-11-20) and conformed to the standards of use of human participants in research as outlined in the Sixth *Declaration of Helsinki*. The participants were informed of the experimental procedures and gave their written consent before any testing was conducted. Also, written informed consent was obtained from the parents or legal guardians of the participants.

### Procedures

#### Session 1

*Anthropometric characteristics.* Body mass (BM in kg) was measured using a digital weight scale with a precision of ± 0.01 kg (Seca 899, SECA, Germany) and height (in m) was assessed using a stadiometer with a precision of ± 1 mm (Seca 213, SECA, Germany).

*Maximal incremental exercise.* The initial power was set between 40 and 80 W during the first 5 min, and then increased by 10–30 W every 3 min, according to age and the expertise level of participants. Each step was separated by a 30-s rest interval in sitting position to drawn an arterialized capillary blood sample (20 μL) from the earlobe at every step toand hence measure the time course of blood lactate concentration ([La] in mmol∙L^−1^). [La] was determined enzymatically using a Biosen C-Line Clinic lactate analyzer (EFK Diagnostics GmbH, Barleben, Germany). Oxygen uptake, carbon dioxide output, and minute ventilation were continuously monitored with a breath-by-breath analyzer (Quark CPET, Cosmed, Italy) to determine the maximal oxygen consumption (V̇O_2max_ in L∙min^−1^). Heart rate (HR in beats∙min^−1^) was continuously recorded with a heart rate monitor (HRM-Dual, Garmin, Kansas, United States A) to determine the end-exercise maximal value (HR_max_ in beats∙min^−1^). The mechanical power output corresponding to V̇O_2max_ (Pa_max_ in W) was also assessed. V̇O_2max_ was considered to be reached when at least two of the following criteria were met: 1) V̇O_2_ leveling-off, 2) maximal respiratory exchange ratio ≥ 1.1, 3) HR_max_ ≥ 95% of the age-predicted HR_max_ (208.6 – 0.7 × age) (Shargal et al., 2015) and 4) [La] higher than 8 mmol∙L^−1^.

#### Session 2

*Rowing ergometer performance.* After a standardized 20 min warm-up at about 130–140 beats∙min^−1^ and two short sprints (10 s) in the last 5 min, all the participants covered the competition distance corresponding to their age category as fast as possible (2,000 m for U18, 1,500 m for U16 and 1,000 m for U14). A 10 min sitting recovery followed the test. Arterialized capillary blood samples (20 μL) were drawn from the earlobe after warm-up ([La]_wp_ in mmol∙L^−1^) and at 1, 3, 5, and 8 min post-exercise to identify maximal blood lactate concentration ([La]_max_ in mmol∙L^−1^). [La] were determined using the same analyzer as in session 1. Blood lactate increase during exercise (∆[La] in mmol∙L^−1^) was obtained by subtracting [La]_wp_ from [La]_max_ (∆[La] = [La]_max_—[La]_wp_). Oxygen uptake was recorded in resting conditions for 3 min before testing (V̇O_2rest_ in L∙min^−1^) and throughout rowing exercise (V̇O_2perf_ in L∙min^−1^ and %V̇O_2perf_ in % of V̇O_2max_). The time to cover the distance (T_perf_ in s) and the mean power output (PO_perf_ in W and %PO_perf_ in % of Pa_max_) were recorded by the electronic timer included in the rowing ergometer device. The total work produced (W_Tot_ in kJ) was subsequently calculated (W_Tot_ = PO_perf_ ∙ T_perf_). Absolute and relative energy amounts derived from aerobic and anaerobic pathways were assessed using the procedure described below.

### Measurements and calculations

#### Absolute and relative amounts of energy released

The energy released from aerobic pathway (E_Ae_ in kJ) was obtained from the accumulated oxygen consumption during exercise (integrated over T_perf_) by subtracting the corresponding integrated V̇O_2rest_.

The energy derived from anaerobic pathways (E_An_ in kJ) was calculated as the sum of the energy released from lactic (E_AnLa_ in kJ) and alactic (E_AnAl_ in kJ) pathways.

E_AnLa_ was estimated from ∆[La], based on an energy equivalent of 3 ml O_2_ Eq.∙kg^−1^ BM for a blood [La] increase of 1 mmol∙L^−1^ (di Prampero, 1981; Ferretti, 2014).

E_AnAl_ was obtained from 1) an energy equivalent of phosphocreatine (PCr) of 16 ml O_2_ Eq.∙kg^−1^ of muscle mass (MM) (Medbø et al., 1988; Lacour, 1990), 2) MM involvement in rowing of 80.0% of total muscle mass (Mader et al., 1988) and 3) a total muscle mass of 53.6, 50.6 and 46.2% of BM in U18, U16 and U14, respectively (Malina, 1969; Malina, 1986).

The total amount of energy released during the individual performance test (E_Tot_ in kJ) was calculated as the sum of E_Ae_ and E_An_.

E_Ae_, E_An_, E_AnLa_, E_AnAl_ and E_Tot_ were expressed in kilojoules (kJ) by assuming that 1 ml O_2_ in the human body yields 21.131 kJ for a respiratory exchange ratio ≥1.0 (Stegemann, 1991).

Based on the absolute values (kJ), the relative contributions of E_Ae_, E_An_, E_AnLa_ and E_AnAl_ were then expressed as a percentage of E_Tot_ (%E_Ae_, %E_An_, %E_AnLa_, %E_AnAl_, respectively).

#### Allometric modeling procedures

*Simple allometric modeling*. The allometric approach is used to remove any dimensional effect on physiological parameters and thereby allow fair comparisons among populations of different body dimensions. As the large range of BM in the studied population (35.6–86.8 kg) may have influenced the estimation of energy released from different metabolic pathways (i.e., E_Ae_, E_An_ and E_Tot_) and the total work produced (W_Tot_), we further investigated the influence of BM on E_Ae_, E_An_, E_Tot_ and W_Tot_ by considering BM as scaling factor using an allometric modeling procedure. The allometric relationships obtained between BM, E_Ae_, E_An_, E_Tot_ and W_Tot_ were based on the general allometric equation (Nevill et al., 1992):

$$y=\;a_{1}\cdot BM^{b_{1}}$$ (1)

where *y* is E_Ae_, E_An_, E_Tot_ and W_Tot_, *a*_*1*_ is the proportionality coefficient associated for each age category, and *b*_*1*_ is the scaling factor associated with BM. The resultant power function ratio *y* ∙ BM^*b1*^ is allegedly free from the confounding influence of BM. To determine *a*_*1*_ and *b*_*1*_, the statistical approach to allometry uses a simple logarithmic transformation as follows:

$$\text{log}\left(y\right)=\;\text{log}\left(a_{1}\right)+b_{1}\cdot \text{log}\left(BM\right)$$ (2)

where *b*_*1*_ is the slope of the linear regression. This slope is calculated by regression analysis, where *b*_*1*_ in the regression output is equal to the scaling factor, and the inverse log of log(*a*_*1*_) is equivalent to the constant *a*_*1*_ in the Eq. 1.

## Statistical analyses

Analyses were performed using OriginPro 2020b software (OriginLab, Massachusetts, United States A). Descriptive statistics were expressed by age category (U18, U16 and U14) as mean ± standard deviation (SD) and 95% confidence interval (lower 95% CI - upper 95% CI). Data were screened for normality of distribution and homogeneity of variances using a Shapiro-Wilk normality test and the Bartlett’s test, respectively. As normality and/or homogenity were not reached, the non-parametric Kruskal-Wallis’ test was used to analyze differences between age categories regarding the investigated mechanical and physiological variables. Mann-Whitney test was used for pairwise comparisons (U18 vs. U16 and U16 vs. U14) when Kruskal-Wallis’ test revealed a significant effect. The effect size and statistical power have also been reported when significant main effects were detected. The effect size was assessed by Hedges’ g $\left(\frac{mean_{1}-mean_{2\;}}{\sqrt{\left(n_{1}-1\right)SD_{1\;}^{2}+\;\left(n_{2}-1\right)SD_{2}^{2}/\;\left(n_{1}+\;n_{2}\;-\;2\right)}}\right)$ ranked as follows: 0.1 = small effect, 0.3 = moderate effect, ≥ 0.5 = large effect (Cohen, 1988). Linear regression models between the parameters were fitted by the least-squares method. The squared Bravais-Pearson correlation coefficient (*r* ^*2*^) of these linear regression models was calculated. In accordance with Hopkins (Hopkins, 2000), the magnitude for squared correlation coefficient was considered as trivial (*r* ^2^ < 0.01), small (0.01 < *r* ^2^ < 0.09), moderate (0.09 < *r* ^2^ < 0.25), large (0.25 < *r* ^2^ < 0.49), very large (0.49 < *r* ^2^ < 0.81), nearly perfect (*r* ^2^ > 0.81) and perfect (*r* ^2^ = 1.0). The statistical significance level was set at 5% (i.e., *p* < 0.05).

## Results

### Participants’ physical and physiological characteristics

Age, height and BM are detailed in Table 1. Maximal heart rate (HR_max_) was similar among the three age groups. Maximal oxygen uptake (V̇O_2max_) was not significantly different between U18 and U16 (Table 1), but 27% lower in U14 than U16 (*g* = 1.80, *p* < 0.001). The power output corresponding to V̇O_2max_ (Pa_max_) was significantly lower in U16 than U18 (− 28%, *g* = 1.07, *p* < 0.05) and in U14 than U16 (−28%, *g* = 1.49, *p* < 0.01).

**TABLE 1:** Participants’ physical characteristics in rowers under 18 years (U18), under 16 years (U16), and under 14 years (U14).

	U18 (*n* = 9)	U16 (*n* = 10)	U14 (*n* = 12)
Age (years)	16.6 ± 0.5 (16.2–17.0)	14.9 ± 0.5 (14.5–15.3)	13.3 ± 0.4 (13.0–13.6)
Height (m)	1.81 ± 0.05 (1.77–1.85)	1.78 ± 0.08^**^ (1.73–1.84)	1.65 ± 0.08 (1.60–1.70)
BM (kg)	72.8 ± 7.2 ^$^ (67.2–78.3)	63.7 ± 7.4^*^ (58.3–69.0)	54.6 ± 9.0 (48.9–60.3)
HR_max_ (beats∙min^−1^)	201 ± 4 (198–205)	200 ± 9 (194–206)	205 ± 8 (200–211)
V̇O_2max_ (L∙min^−1^)	4.6 ± 0.4 (4.3–4.8)	4.1 ± 0.5^***^ (3.8–4.5)	3.0 ± 0.7 (2.5–3.4)
Pa_max_ (W)	277 ± 29 ^$^ (254–299)	240 ± 35^**^ (215–265)	172 ± 49 (141–203)

Data are means ± SD (lower 95% CI, upper 95% CI). ^$^ and ^$$$^: significantly different between U18 and U16 at *p* < 0.05 and *p* < 0.001, respectively. *, ** and ***: significantly different between U16 and U14 at *p* < 0.05, *p* < 0.01 and *p* < 0.001, respectively. BM, body mass; HR_max_, maximal heart rate; V̇O_2max_, maximal oxygen consumption; Pa_max_, mechanical power corresponding to V̇O_2max_.

### Rowing ergometer performance

Oxygen uptake at rest (V̇O_2rest_) was 0.58 ± 0.08 (0.53–0.64) L∙min^−1^ for U18, 0.56 ± 0.07 (0.51–0.61) L∙min^−1^ for U16 and 0.47 ± 0.07 (0.42–0.51) L∙min^−1^ for U14. The mean time (T_perf_) to cover 2,000 m was 7 min 02 ± 18 s (6 min 48 s–7 min 16 s), 5 min 28 ± 18 s (5 min 15 s–5 min 40 s) for 1,500 m, and 4 min 06 ± 25 s (3 min 50 s–4 min 21 s) for 1,000 m. As indicated in Table 2, the mean power output sustained during the rowing ergometer exercise (PO_perf_) was not significantly different between U18 and U16 but significantly lower in U14 than U16 (−27%, *g* = 1.40, *p* < 0.01). Relative to Pa_max_, %PO_perf_ was not significantly different among the three age categories. The mean oxygen consumption sustained during the race was significantly higher in U18 than U16 (+13%, *g* = 1.11, *p* < 0.01) and in U16 than U14 (+26%, *g* = 1.56, *p* < 0.001). However, relative to V̇O_2max,_ %V̇O_2perf_ was not significantly different among the three age categories. [La]_wp_ was similar among the three age categories. While no significant difference was observed for [La]_max_ and ∆[La] between U18 and U16, [La]_max_ and ∆[La] were significantly lower in U14 than U16 (−15%, *g* = 1.42, *p* < 0.01 and −12%, *g* = 1.62, *p* < 0.01, respectively).

**TABLE 2:** Performance and physiological characteristics obtained during the rowing ergometer test over 2,000 m for rowers under 18 years (U18), 1,500 m for rowers under 16 years (U16) and 1,000 m for rowers under 14 years (U14).

	U18 (*n* = 9)	U16 (*n* = 10)	U14 (*n* = 12)
Distance (m)	2,000	1,500	1,000
PO_perf_ (W)	301 ± 39 (270–331)	273 ± 43^**^ (242–304)	199 ± 55 (164–234)
%PO_perf_ (%Pa_max_)	109 ± 7 (104–113)	114 ± 11 (106–122)	117 ± 112 (109–124)
V̇O_2perf_ (L∙min^−1^)	4.5 ± 0.4 ^$$^ (4.2–4.8)	3.9 ± 0.5^***^ (3.6–4.2)	2.9 ± 0.7 (2.5–3.3)
%V̇O_2perf_ (%V̇O_2max_)	98 ± 3 (96–101)	95 ± 3 (92–97)	98 ± 7 (94–103)
[La]_wp_ (mmol∙L^−1^)	1.4 ± 0.6 (1.0–1.8)	1.6 ± 0.6 (1.2–2.0)	2.0 ± 0.8 (1.5–2.5)
[La]_max_ (mmol∙L^−1^)	17.6 ± 2.8 (15.8–19.5)	16.0 ± 1.5^**^ (15.1–16.9)	13.5 ± 1.7 (12.6–14.5)
∆[La] (mmol∙L^−1^)	16.3 ± 2.5 (14.6–17.9)	14.4 ± 1.4^**^ (13.5–15.3)	11.5 ± 2.0 (10.4–12.7)

Data are means ± SD (lower 95% CI, upper 95% CI). ^$$^: significantly different between U18 and U16 at *p* < 0.01. ** and ***: significantly different between U16 and U14 at *p* < 0.01 and *p* < 0.001, respectively. PO_perf_, mean power output; %PO_perf_, PO_perf_ relative to Pa_max_; V̇O_2perf_, mean oxygen consumption sustained; %V̇O_2perf_, V̇O_2perf_ relative to V̇O_2max_; [La]_wp_, lactate concentration after warm-up; [La]_max_, post-exercice maximal lactate concentration; ∆[La], lactate increase during exercise.

### Absolute amount of work produced and energy released

The total work produced during the rowing ergometer test (W_Tot_) was significantly lower in U16 than U18 (−30%, *g* = 3.69, *p* < 0.001) and in U14 than U16 (−46%, *g* = 4.49, *p* < 0.001).

**FIGURE 1:**
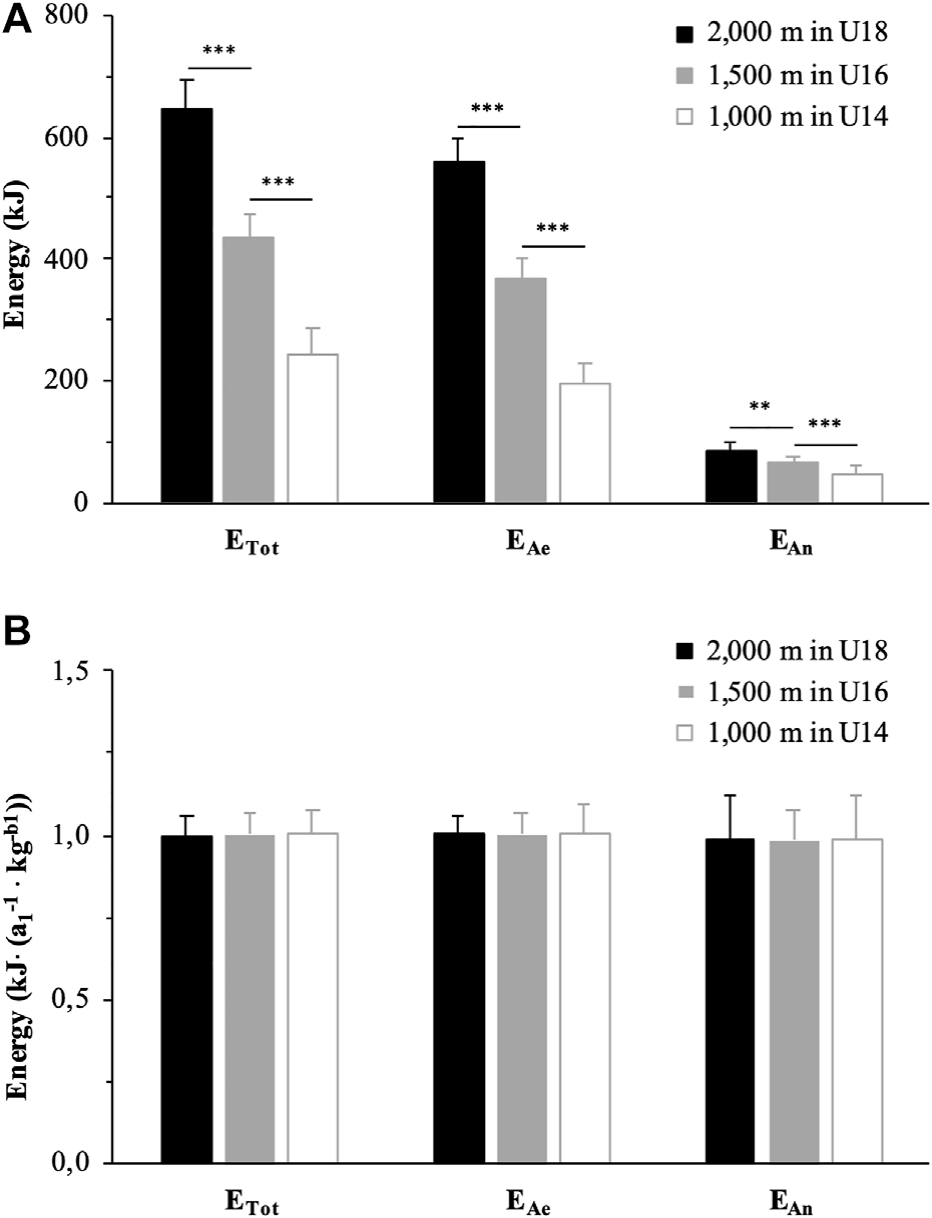
Quantity of energy released from metabolic pathways during the exhaustive rowing ergometer exercise over 2,000 m in rowers under 18 years (U18, black), 1,500 m in rowers under 16 years (U16, grey) and 1,000 m in rowers under 14 years (U14, white) expressed in absolute values **(A)** and allometrically scaled for body mass **(B)**. ** and ***: significantly different at *p* < 0.01 and *p* < 0.001, respectively. E_Tot_: total amount of energy released; E_Ae_: amount of energy released from aerobic metabolism; E_An_: amount of energy released from anaerobic pathways; *a*_*1*_ and *b*_*1*_: simple allometric model parameters.

The amounts of energy released from each metabolic pathway, expressed in absolute value, are reported in Table 3. E_Ae_ and E_An_ were significantly lower in U16 than U18 (−34%, *g* = 5.18, *p* < 0.001 and -20%, *g* = 1.39, *p* < 0.01, respectively) and in U14 than U16 (−47%, *g* = 4.95, *p* < 0.001 and −28%, *g* = 1.77, *p* < 0.001, respectively). E_AnLa_ and E_AnAl_ were also significantly lower in U16 than U18 (−22%, *g* = 1.37, *p* < 0.05 and −10%, *g* = 1.17, *p* < 0.05, respectively) and in U14 than U16 (−31%, *g* = 1.81, *p* < 0.001 and -11%, *g* = 1.03, *p* < 0.05, respectively). As a result, E_Tot_ was significantly lower in U16 than U18 (−33%, *g* = 4.56, *p* < 0.001) and in U14 than U16 (−44%, *g* = 4.37, *p* < 0.001). E_Ae,_ E_An_ and E_Tot_ are illustrated by race distance (or age category) in Figure 1A.

**TABLE 3:** Amount of energy released from metabolic pathways and mechanical work produced during the rowing ergometer test over 2,000 m for rowers under 18 years (U18), 1,500 m for rowers under 16 years (U16) and 1,000 m for rowers under 14 years (U14).

	U18 (*n* = 9)	U16 (*n* = 10)	U14 (*n* = 12)
Distance (m)	2,000	1,500	1,000
E_Ae_			
Absolute (kJ)	560 ± 37 ^$$$^ (536–584)	367 ± 34^***^ (346–388)	196 ± 33 (177–215)
Relative (kJ∙min^−1^)	80 ± 9^*^ (74–86)	68 ± 9^***^ (62–73)	49 ± 12 (42–56)
E_An_ (kJ)			
Absolute (kJ)	84 ± 16 ^$$^ (74–96)	67 ± 8^***^ (62–71)	48 ± 12 (41–54)
Relative (kJ∙min^−1^)	12 ± 3 (10–14)	12 ± 2 (11–13)	12 ± 4 (10–14)
E_AnLa_ (kJ)			
Absolute (kJ)	74 ± 15 ^$^ (65–84)	58 ± 7.0^***^ (54–62)	40 ± 11 (34–46)
Relative (kJ∙min^−1^)	11 ± 2 (9–12)	11 ± 2 (10–12)	10 ± 3 (8–12)
E_AnAl_ (kJ)			
Absolute (kJ)	10 ± 1 ^$^ (9–11)	9 ± 1^*^ (8–9)	8 ± 1 (7–8)
Relative (kJ∙min^−1^)	1.4 ± 0.2 (1.3–1.5)	1.6 ± 0.3 (1.4–1.7)	1.8 ± 0.5 (1.6–2.1)
E_Tot_ (kJ)			
Absolute (kJ)	645 ± 47 ^$$$^ (614–676)	434 ± 40^***^ (409–458)	244 ± 42 (220–268)
Relative (kJ∙min^−1^)	92 ± 10 ^$^ (85–99)	80 ± 11^**^ (73–87)	61 ± 15 (52–69)
W_Tot_ (kJ)			
Absolute (kJ)	126 ± 11 ^$$$^ (119–134)	89 ± 9^***^ (93–95)	48 ± 9 (43–53)
Relative (kJ∙min^−1^)	18 ± 2 (17–20)	16 ± 3^**^ (15–18)	12 ± 3 (10–14)

Data are means ± SD (lower 95% CI, upper 95% CI). ^$^, ^$$^ and ^$$$^: significantly different between U18 and U16 at *p* < 0.05, *p* < 0.01 and *p* < 0.001, respectively. * and ***: significantly different between U16 and U14 at *p* < 0.05 and *p* < 0.001, respectively. E_Ae_: amount of energy released from aerobic metabolism; E_An_: amount of energy released from anaerobic pathways; E_AnLa_: amount of energy released from lactic anaerobic metabolism; E_AnAl:_ amount of energy released from alactic anaerobic metabolism; E_Tot_: total amount of energy released; W_Tot_: total mechanical work produced.

### Allometric modeling

BM was positively and significantly correlated to E_Tot_ (*r* ^2^ = 0.67, *p* < 0.001) and W_Tot_ (*r* ^2^ = 0.66, *p* < 0.001).

Allometric scaling exponents obtained from Eq. 2 are detailed in Table 4.

**TABLE 4:** Allometric coefficients resulting from simple model associated with the energy amount derived from aerobic and anaerobic pathways and total mechanical work produced.

**Simple allometric model *y* = *a*_*1*_ · BM^*b1*^**					
	**(U18)**	***a*_*1*_ (U16)**	**(U14)**	***b*_*1*_**	***r* ^2^**
E_Ae_	21.7	15.7	9.4	0.76	0.98
E_An_	0.50	0.46	0.39	1.20	0.86
E_Tot_	18.0	13.5	8.6	0.84	0.98
W_Tot_	3.64	2.86	1.73	0.83	0.97

E_Ae_: amount of energy released from aerobic metabolism; E_An_: amount of energy released from anaerobic pathways; E_Tot_: total amount of energy released; W_Tot_: total mechanical work produced; *a*_*1*_ and *b*_*1*_: simple allometric model parameters.

When scaled for BM^*b1*^, E_Ae_, E_An_ and E_Tot_ were not significantly different between 2,000 m for U18, 1,500 m for U16, and 1,000 m for U14 (*p* > 0.05) (Figure 1B). Similarly, no significant difference was observed for W_Tot_ scaled for BM^*b1*^ between the three race distances.

### Correlations between mechanical and energetic parameters

**FIGURE 2:**
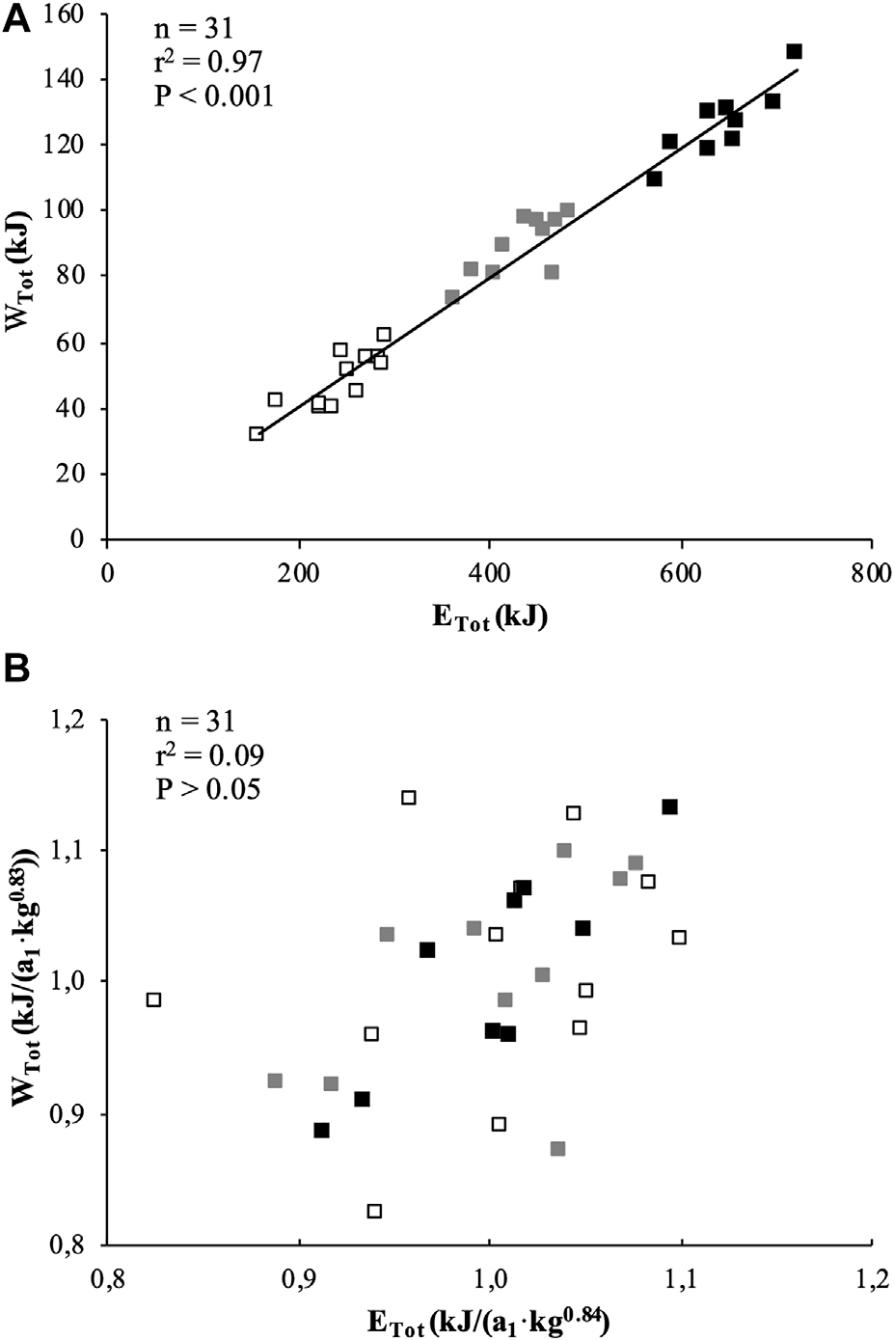
Correlations between total mechanical work (W_Tot_) and total energy released (E_Tot_) during the exhaustive rowing ergometer exercise expressed in absolute values **(A)** and allometrically scaled for body mass **(B)**. Black squares represent U18, grey squares represent U16 and white squares represent U14 rowers.

W_Tot_ was significantly correlated to E_Tot_ when expressed in absolute value (Figure 2A) or scaled for BM (Figure 2B).

### Relative energy contributions

**FIGURE 3:**
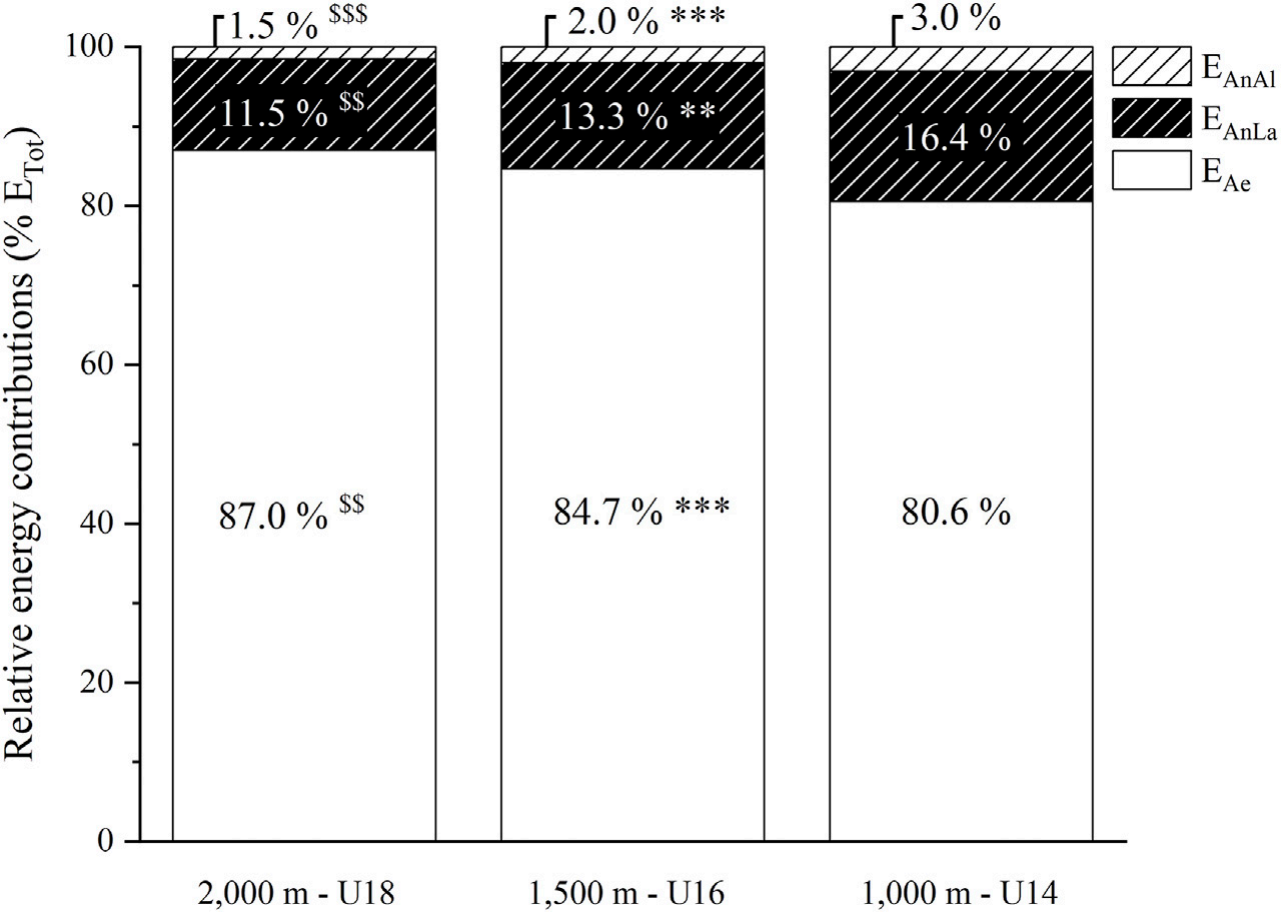
Relative aerobic and anaerobic (alactic and lactic) energy contributions (%E_Ae_ and %E_AnAl_ and %E_AnLa_, respectively) expressed as a percentage of total energy released (E_Tot_) during the exhaustive rowing ergometer exercise over 2,000 m in rowers under 18 years (U18, *n* = 9), 1,500 m in rowers under 16 years (U16, n = 10) and 1,000 m in rowers under 14 years (U14, n = 12). ^$$^ and ^$$$^: significantly different between U18 and U16 at *p* < 0.01 and *p* < 0.001, respectively. ** and ***: significantly different between U16 and U14 at *p* < 0.01 and *p* < 0.001, respectively.

As illustrated by Figure 3 **,** %E_Ae_ was lower in U16 than U18 (-3.4% *g* = 1.59, *p* < 0.01) and lower in U14 than U16 (−4.1% *g* = 1.72, *p* < 0.001), while %E_An_ was higher in the same proportions in U16 than U18 and in U14 than U16. %E_AnLa_ and %E_AnAl_ were also significantly higher in U16 than U18 (+1.8%, *g* = −1.30, *p* < 0.01 and +0.5%, *g* = −3.31, *p* < 0.001, respectively) and in U14 than U16 (+3.1%, *g* = −1.30, *p* < 0.01 and +1.1%, *g* = −4.95, *p* < 0.001, respectively).

## Discussion

The present study aimed to compare the relative aerobic (%E_Ae_) and anaerobic (%E_An_) energy contributions into total energy supply between 2,000 m in U18 rowers, 1,500 m in U16 rowers, and 1,000 m in U14 rowers. The results of the present study did not confirm our initial hypothesis since %E_Ae_ and %E_An_ were significantly different among the race distances, and thereby the age categories. However, %E_An_ in U18, U16 and U14 were found to be in the range of values previously found in adult rowers over the 2,000 m Olympic distance (12–30%). Therefore, on a practical level, the results of the present study seem to confirm the strategy implemented by the French rowing federation to reduce the competition distance in the younger age categories to prepare them progressively to the physiological requirements encountered over the 2,000 m Olympic distance.

### Physical and physiological characteristics

Rowing performance parameters have been mostly investigated in adult rowers, particularly in high-level athletes (Ingham et al., 2002; Bourdin et al., 2004; Mikulić, 2011; Bourdin et al., 2017). Little information is available about specific rowing performance in competitive 12- to 17-year-old rowers. To the best of our knowledge, only three studies reported data that can be explicitly compared to our outcomes (Russell et al., 1998; Mikulić and Ružić, 2008; Giroux et al., 2017). For instance, in U18, rowing performance was 4.8% lower in our rowers compared to those evaluated by Russell et al. (1998) on 2,000 m (7 min 02 ± 18 s vs. 6 min 43 ± 16 s, respectively). In U16, our data are comparable to those reported by Giroux et al. (2017) on 1,500 m (5 min 28 ± 18 s vs. 5 min 26 ± 20 s, respectively). In U14, rowing performance was 2.8% higher in our rowers compared to those evaluated by Mikulić and Ružić (2008) on 1,000 m (4 min 06 ± 24 s vs. 4 min 13 ± 24 s, respectively). In addition, V̇O_2max_ measured in our rowers (Table 1) were comparable to those reported by Russell et al. (1998) and Mikulić and Ružić (2008) in U18 and U14 (4.6 ± 0.4 and 2.8 ± 0.5 L min^−1^, respectively). Taken together, these data suggest that rowing performance and physical fitness were comparable among our French competitive rowers and U18 Australian and U14 Croatian national-level rowers.

### Young rowers’ capacity to perform an exhaustive rowing exercise

Despite their young age, the rowers of the present study were able to sustain exercise intensity between 95 and 98% of their V̇O_2max_ for ∼7 min over the 2,000 m in U18 and for ∼4 min over the 1,000 m in U14 (Table 2). This finding is consistent with the data reported by Gillies and Bell (2000), showing a mean exercise duration of 7 min 06 s sustained at 94% of V̇O_2max_ over the 2,000 m rowing ergometer performance in adult rowers. Similarly, mean mechanical power outputs (PO_perf_) sustained over the 2,000 in U18, 1,500 in U16 and 1,000 m in U14 were found to be 109, 114 and 117% of Pa_max_, respectively. These remarkable results are closely comparable to those reported in high-level rowers performing an exhaustive 2,000 m exercise on a rowing ergometer (113% of Pa_max_) (Bourdin et al., 2004). Thus, our data show for the first time that young competitive rowers were able to maintain supra-maximal intensities (i.e., higher than Pa_max_) during prolonged exercise (from ∼4 to 7 min). This finding may be associated with their capacity to highly stimulate glycolytic metabolism, as evidenced by the high values of [La]_max_ reported at the end of the 2,000, 1,500 and 1,000 m exhaustive exercises (Table 2).

### Estimated amounts of energy released

Our results showed that the absolute amount of energy released from aerobic (E_Ae_) and anaerobic (E_An_) energy pathways decreased with the reduction of rowing competition distances (Table 3). Comparatively, using a similar estimation method, de Campos Mello et al., 2009 reported values of E_Ae,_ E_An_ and E_Tot_ in adult rowers comparable to those obtained in our U18 rowers (563 vs. 560 kJ for E_Ae_, 106 kJ vs. 84 kJ for E_An_ and 674 vs. 645 kJ for E_Tot_).

From the method of accumulated oxygen deficit, Diry et al. (2020) showed that the quantity of anaerobic energy released during a 60 s ‘all-out’ exercise in young competitive rowers was positively influenced by their body dimensions. In the present study, an original allometric approach was used to remove the dimensional effect of BM on the absolute quantity of aerobic and anaerobic energy supplied during the 2,000-, 1,500-, and 1,000-m rowing exercises. The current results show that the BM-specific allometric coefficients (i.e., *b*_*1*_ in Eq. 1 and Table 4) are lower than 1 (0.76 for E_Ae_ and 0.84 for E_Tot_), suggesting that E_Ae_ and E_Tot_ would increase in lower proportions than BM. Conversely, the BM-specific allometric coefficient was 1.20 for E_An_ (Table 4) suggesting that E_An_ would increase in higher proportion than BM. Interestingly, the BM-specific allometric coefficient associated to E_An_ (i.e., 1.20) is comparable to one previously reported by Maciejewski et al. (2016b) regarding power output during a modified rowing Wingate test in U16 rowers (i.e., 1.24).

### Relationships between mechanical and physiological parameters

In the present study, the total work (W_Tot_) produced over 2,000, 1,500, and 1,000 m was found to be closely correlated to the total energy (E_Tot_) expended over the corresponding distances. Our results show that W_Tot_ accounted for 98% in the variation of E_Tot_ (Figure 2A). This significant result clearly illustrates that, despite the assumptions made to estimate the relative aerobic and anaerobic contibutions (see *Methods section*), a very close relationship persists between the mechanical (W_Tot_) and physiological (E_Tot_) parameters. In addition, when calculating the rowing gross efficiency (in %), as E_Tot_ divided by W_Tot_, there were no significant differences among categories: 19.5, 20.5 and 19.7% for U18, U16 and U14, respectively. Interestingly, these results are in the same range as those of highly trained adult rowers (i.e., 18.5%) (Bourdin et al., 2004), and show that young competitive rowers are able to reach the same rowing gross efficiency as their adult elite counterparts.

However, further analysis shows that BM would explain 66 and 67% of the variations in W_Tot_ and E_Tot_, respectively. The allometric method used to remove the effects of BM on W_Tot_ and E_Tot_ shows that these two parameters have similar BM-specific allometric coefficients (Table 4), and they are no longer correlated when scaled for their respective allometric coefficients (*r* ^2^ = 0.09) (Figure 2B), suggesting that BM would have a very strong influence (91%) on this relationship. Therefore, our outcomes clearly confirm 1) the interest and accuracy of the method used in the present study for estimating the energy amounts derived from aerobic and anaerobic pathways and 2) the need to use allometric modeling to appreciate the influence of BM on W_Tot_ and E_Tot_.

### Relative anaerobic energy contribution

The outcomes of the present study confirm that anaerobic metabolism is a non-negligible energy source during rowing competitions, whatever the considered competition distances (Figure 3). Although a statistical difference exists, the estimated relative anaerobic contributions over the three distances (13.0% for 2,000 m, 15.3% for 1,500 m and 19.4% for 1,000 m) were found to be relatively comparable from a practical perspective. Because these values are estimated and not directly measured, we cannot exclude approximations inherent to our calculation methods (*vide supra Method section*). However, it is worth noting that our estimations are similar to those previously reported in the literature for adult rowers covering the distance of 2,000 m. For example, using a similar method to ours, de Campos Mello et al., 2009 reported that the anaerobic pathways provided about 16% of the total energy expended over a 2,000 m rowing exercise in national-level adult rowers. Similarly, over the Olympic distance, Secher et al. (Secher et al., 1982; Secher, 1983) estimated from the oxygen debt method that about 14% of the energy expended was of anaerobic origin in elite rowers. These results were confirmed by Pripstein et al. (Pripstein et al., 1999), using the accumulated oxygen deficit method in university adult rowers (12%). The similarities between our results and those of previous studies (Secher et al., 1982; Secher, 1983; Pripstein et al., 1999; de Campos Mello et al., 2009) are consistent despite the 1) differences in rowing exercise duration between child, adolescent and adult rowers and, 2) heterogeneous muscle mass between populations, which is known to influence anaerobic energy supply (*vide supra*) (Bangsbo et al., 1993; Gastin, 2001; Diry et al., 2020).

## Conclusion

Despite significant differences in %E_Ae_ and %E_An_ between the race distances (2,000 m, 1,500 m and 1,000 m), %E_An_ were found to be in the range of values previously found in adult rowers over the 2,000 m Olympic distance (12–30%). Therefore, on a practical level, the results of the present study seem to confirm the strategy implemented by the French rowing federation to reduce the competition distance in the younger age categories to prepare them progressively to the physiological requirements encountered over the 2,000 m Olympic distance.

## Practical applications

Olympic rowing is currently facing a considerable increase in results density. While certain areas for improving performance still require special attention (e.g., setting the rowers up in their boats, using new techniques to promote recovery, etc*.*), the area aimed at better preparing young rowers for the demands of the top level is by far the most under-exploited in rowing, despite the considerable benefits it is likely to bring. From a physiological point of view, this improvement in the preparation of young rowers requires a better knowledge of the energy requirements supplied during rowing competitions.

Because the aerobic pathway is the main source in the total energy supply, whatever the competition distances, the development of V̇O_2max_ should be the common thread in the training program for the youngest rowers, particularly through alternating continuous and intermittent training sessions. In the present study, we showed that young rowers are able to maintain an intensity comprised between 95 and 98% of V̇O_2max_ during a rowing ergometer competition. Based on the results of Leclair et al. (2011), showing that anaerobic capacity would promote the V̇O_2max_ maintenance time in children, the development of anaerobic pathways in younger rowers could be an interesting issue of work for rowing coaches, which however should be used carefully and sparingly to avoid impairment in the development of the aerobic pathway.

Finally, beyond metabolic considerations, it is interesting to underline that reducing the competition distance to 1,500 m for U16 and to 1,000 m for U14 would allow 1) to reinforce the motivational aspects to reduce the young rowers’ drop-out rate during the first years and 2) to decrease the time difference between engaged crews and, *in fine*, to keep rowing races more attractive. From a technical point of view, shortening competitive distance can also allow to help young rowers to maintain high technical standards to 1) optimize boat speed and 2) prevent the risk of injury that can occur if the technical level deteriorates with the lengthening of the competitive distances.

## Data Availability

The raw data supporting the conclusion of this article will be made available by the authors, without undue reservation.
